# Dataset describing problem factors of partnering relationships in the Malaysian construction industry

**DOI:** 10.1016/j.dib.2022.108733

**Published:** 2022-11-08

**Authors:** Eric Yao, Yet-Mee Lim, Choi-Meng Leong, Wai-Chow Lee, Chuen-Khee Pek

**Affiliations:** a UCSI Graduate Business School, UCSI University, Malaysia; b Swiss Institute of Management and Innovation, Zug, Switzerland; c Faculty of Business and Management, UCSI University, Malaysia

**Keywords:** Main contractor, Subcontractor, Project owner, Project consultant, External environment

## Abstract

The dataset indicates the problem factors which may influence the development of partnering relationship between two key project parties - the main contractor and the subcontractor. A total of 53 problem factors were identified and were clustered into five categories. An online questionnaire was used to elicit the viewpoints of these key parties with regards to the extent to which the factors have an impact on the partnering relationship in the Malaysian construction industry. The target respondents were managers and engineers of various construction companies in Malaysia with the main contractors or subcontractors who have projects in Kuala Lumpur, Shanghai and New Delhi. Both descriptive and quantitative analysis approaches were used to present the data. Relative Importance Index method was used to determine the top challenges to building favourable partnering relationships; and Partial Least Squares Structural-Equation Modelling (PLS-SEM) analysis was conducted to examine the role played by each cluster of factors in explaining the quality of partnering relationship between the main contractors and subcontractors. This data set would assist practitioners to work on the major problem factors to improve the quality of partnering relationship between the key participants in their present and future construction projects. By eliminating or minimizing these problem factors, practitioners will contribute significantly to effective supply chain management in the construction industry. The data would be of value to academics and industry professionals involved in the construction business domestically and internationally.

**Specifications table**

**Table:** 

Subject	Supply Chain Management
Specific subject area	Partnering Relationships in Construction Industry
Type of data	Excel File SmartPLS version 3.2.8.
How the data were acquired	The data was collected from the targeted respondents through online survey, which was distributed via online platform, namely Google Forms. Data were coded in Excel file format and processed via SmartPLS version 3.2.8.
Data format	Raw Analyzed Filtered
Description of data collection	The dataset were collected from managers and engineers of various construction companies in Malaysia with the main contractors or subcontractors who have projects in Kuala Lumpur, Shanghai and New Delhi using purposive sampling technique. Data were gathered from 144 respondents. We excluded the non-targeted respondents, leaving 124 respondents.
Data source location	City/Town/Region: Kuala Lumpur, Shanghai, New Delhi Country: Malaysia, China, India
Data accessibility	Repository name: Mendeley Data Data identification number: 10.17632/hh8h42yprg.2 Direct URL to data: https://data.mendeley.com/datasets/hh8h42yprg

## Value of the data

• The data collected help to identify the problem factors leading to the adversarial relationships among the construction industry players as a result of conflicting interests in the supply chain.
• The data will assist practitioners to focus on the major problem factors which have existed between participants in the construction industry in their present and future projects.
• Specifically, the data indicate that problems related to the subcontractors (substandard quality of work and non-adherence to the conditions of the contract) negatively affect the main and subcontractor partnering relationship, which in turn, affects the performance of the construction projects.
• The data imply that careful selection of subcontractors is important. Stringent selection of subcontractors and close monitoring of their performance are critical in enhancing successful project implementation with the aim of maximizing profit and minimizing risks.
• The data will raise the awareness of the stakeholders such as Malaysian private construction companies (e.g. Gamuda Berhad, UEM Group Berhad etc.), Ministry of Works, various agencies under Ministry of Works such as The Construction Industry Development Board (CIDB) of Malaysia, and research committees (e.g. Universiti Malaya, Universiti Kebangsaan Malaysia etc.) with regards to the problem factors in the partnering relationships while undertaking projects overseas.
• Finally, the dataset serves as the baseline for academic researchers to compare and contrast the partnering problem factors which will be of great help to the industry professionals in the domestic (e.g. Malaysian Institute of Property & Facility Managers, and The Building Management Association of Malaysia) and international construction business (e.g. International Facility Management Association).

## Objective

Quality supply chain management practises are critical in the business industry [1,2,3]. However, few studies have examined the entirety of a partnered relationship from the perspectives of both sides especially in the construction industry. Furthermore, it is not addressed how problem elements and partnering relationships relate to the effective completion of building projects. The adoption of partnership relationships in supply chain management has been hampered by several discrepancies despite practitioners' realisation that these relationships must be long-lasting and mutually beneficial. These inconsistencies are caused by a number of participant-related issues. The dataset aims to reveal the participant-related issues in the construction industry.

## 1 Data description

The dataset [4] comprises of the problem factors leading to conflicts between two parties in the construction industry, specifically the problem factors impeding the development of partnering relationships. Fifty-three (53) problem factors were identified from the literature and were grouped into five categories: (1) factors related to the project owner, (2) factors related to the main contractor, (3) factors related to the subcontractor, (4) factors related to the consultant, and (5) factors related to the external environment. In addition, the dataset also presents the relationship indicator between contractor and subcontractor, which reflects the level of partnering or quality relationship between main contractor and subcontractor. Data were coded in Excel file format and processed via SmartPLS version 3.2.8. Table 1 presents the 53 problem factors by category as well as the relationship indicator. Pilot testing was also conducted and therefore MC21 and PO7 were excluded from the questionnaire for final data collection due to the low loading values. The data is described and tabulated from Tables 2 to 6.

**Table 1:** Problem factors by category

Factors Related to the Main Contractor	
MC1	Main contractor does not fully understand contract content
MC2	Assigning part of the works to new subcontractor, without the consent of original subcontractor
MC3	Failure to provide the subcontractors with essential services such as electricity, water, etc
MC4	Failure of the main contractor to use the insurance in case of injury of subcontractor's labour
MC5	Failure to provide proper security at the site for plants and equipment
MC6	Main contractor provides off-site location for storage of material
MC7	Main contractor personnel are absent frequently at site
MC8	Involvement of the main contractor in several projects at the same time
MC9	Scheduling conflicts between the contractor and subcontractor
MC10	Scheduling conflicts among the subcontractors
MC11	Inappropriate arrangement to the subcontractors works
MC12	Low experience and low capability of the main contractor
MC13	Awarding the specific subcontractor because of his low price only
MC14	Main contractor's financial problem
MC15	Delay in contract progress payments
MC16	Interruptions and termination of work by the contractor
MC17	Delay in shop drawings and sample material approval
MC18	Delay by the main contractor in providing the necessary materials to the subcontractor
MC19	Providing low-quality materials that result in low-quality workmanship
MC20	Delay of the main contractor in submitting the formal documents to the supervision staff, which leads to delay in implementing the works
MC21	Failure to provide complete clarifications of drawings to the subcontractor
**Factors Related to the Subcontractor**	
SC1	Non-adherence of the subcontractor to the time schedule
SC2	Delay of the works behind the time schedule
SC3	Failure to save and take care of the materials and equipment at site
SC4	Exhausting the plant and resources of the main contractor
SC5	Partnering the works to another subcontractor without getting approval of main contractor
SC6	Subcontractor personnel are absent frequently at site
SC7	Subcontractor involves in several projects at the same time
SC8	Neglecting the instructions of the main contractor
SC9	Subcontractor fail to deploy sufficient workers at site
SC10	Non-adherence to the conditions of the contract
SC11	Lack of quality work during construction
SC12	Lack of experience and ability of the subcontractor in similar projects
SC13	Subcontractor fail to deploy sufficient equipment and machinery at site
SC14	Neglecting the safety measures
SC15	Changes in material and labour costs before completion
**Factors Related to the Project Owner**	
PO1	Awarding the tender to the contractor with lowest price
PO2	Delay in providing complete information such as additional drawings, benchmarks, set-backs etc.
PO3	Giving instructions to the subcontractor directly without consulting the main contractor
PO4	Objection of the owner on the implementation method used by the subcontractor
PO5	Poor variation management
PO6	Delay in releasing payments to the main contractor
PO7	Short period allowed for implementing the project
**Factors Related to the Project Consultant**	
CO1	inaccurate among the tender documents
CO2	Incomplete work-drawing and technique specifications
CO3	Delay of approval of the finished work
CO4	Delay in approving materials samples and shop drawings
CO5	lack of experience of the consultant's team
**Factors Related to the External Environment**	
EE1	Shortage of construction materials and equipment in the market
EE2	Weather conditions
EE3	Geological problems on the site
EE4	Breach of the contract due to project termination
EE5	Closing the commercial border crossings
**Quality of Partnering Relationship**	
RI1	Mutual objectives were established to benefit both parties
RI2	Gain and pain sharing was agreed between the parties
RI3	A high degree of trust exist between parties
RI4	Parties did not blame each other when problems occurred
RI5	Both parties work jointly together
RI6	Communication between the parties was open and effective
RI7	The problem solving mechanism between the parties was effective
RI8	Risk allocation between the parties was clear and fair
RI9	Performance was reviewed and fed back on a regular basis
RI10	Improvement was continuously made during the project

**Table 2:** Profile of the respondents

Profile	Category	Frequency	Percentage
**Type of Project (PT)**	Residential	18	15%
	Non-Residential (Office, Factory etc.)	29	23%
	Infrastructure (Railway, Road, Tunnel etc.)	72	58%
	Social Amenity (Hospital, Campus etc.)	5	4%
**Types of Project Owner (PO)**	Private Limited company	13	10%
	Public limited company	37	30%
	Government	74	60%
**Role of Respondent (RR)**	Main Contractor	73	59%
	Subcontractor	51	41%
**Working Experiences in Construction Sector (WE)**	1-5 years	15	12%
	6-10 years	35	28%
	11-15 years	46	37%
	16-20 years	20	16%
	More than 20 years	8	6%
**Country of Project Located (PL)**	Malaysia	38	31%
	China	44	35%
	India	42	34%
**Project Scheduled Duration (PS)**	0-12 months	13	10%
	12-36 months	70	56%
	36-60 months	38	31%
	60-120 months	3	2%
**Project Budget (USD) (PB)**	Between 120,000-240,000	11	9%
	Between 240,000-1.2 Million	10	8%
	Between 1.2 Million - 2.4 Million	18	15%
	Between 2.4 Million - 24 Million	29	23%
	Between 24 Million - 120 Million	37	30%
	Between 120 Million - 240 Million	11	9%
	More than 240 Million	8	6%

Table 2 presents the profile of the respondents, which comprises the type of project, types of project owners, role of respondent, working experiences in construction sector, country of project located and project budget. Table 3 shows the top 10 problem factors relating to the main contractors and the subcontractors generated by using relative important index (RII) equation. There are 11 problem factors in this top 10 list due to the tie of two factors in rank 7. Five of these 11 problem factors are related to the main contractor and five are related to the subcontractor. Only one problem factor is related to the project owner.

**Table 3:** Top 10 Ranking of Problem Factors

Problem Factor	Related to	Relative Important Index (RII)	Rank
Main contractors’ financial problem	Main contractor	0.868	1
Lack of quality work during construction	Subcontractor	0.861	2
Delay in contract progress payments	Main contractor	0.856	3
Interruptions and termination of work by the contractor	Main contractor	0.855	4
Delay in releasing payments to the main contractor	Project Owner	0.847	5
Awarding the specific subcontractor because of his low price only	Main contractor	0.834	6
Providing low-quality materials that result in low-quality workmanship	Main contractor	0.823	7
Non-adherence to the conditions of the contract	Subcontractor	0.823	7
Delay of the works behind the time schedule	Subcontractor	0.813	8
Lack of experience of the subcontractor in similar projects	Subcontractor	0.81	9
Fail to deploy sufficient workers at site	Subcontractor	0.808	10

Table 4 presents the validity and reliability checking of the data. Factors with loadings of more than 0.70 were retained in the research model and those with less than 0.70 were deemed not reliable and hence, omitted. Cronbach's alpha values and composite reliability (CR) of the factors are all above 0.70 indicating that the items assigned to the respective constructs are reliable. The extracted average variances (AVEs) are all above 0.50 which shows that convergent validity is established in the measurement model [5]. Table 5 shows that the heterotrait-monotrait (HTMT) values are all less than 0.85 indicating that discriminant validity is also established (Hair et al., 2017).

**Table 4:** Composite reliability and average variance extracted

Constructs	Items	Loadings	Cronbach's Alpa	Composite Reliability (CR)	Average Variance Extracted (AVE)
Consultant	CO1	0.874	0.910	0.932	0.732
	CO2	0.875			
	CO3	0.877			
	CO4	0.865			
	CO5	0.782			
Environmental	EE4	0.952	0.792	0.900	0.818
	EE5	0.854			
Main contractor	MC16	0.780	0.776	0.869	0.689
	MC17	0.868			
	MC18	0.840			
Project owner	PO2	0.806	0.817	0.872	0.576
	PO3	0.739			
	PO4	0.802			
	PO5	0.735			
	PO6	0.709			
Quality of partnering relationship	RI10	0.778	0.928	0.941	0.665
	RI2	0.733			
	RI3	0.831			
	RI5	0.843			
	RI6	0.802			
	RI7	0.861			
	RI8	0.808			
	RI9	0.859			
Subcontractor	SC10	0.834	0.759	0.886	0.796
	SC11	0.947

**Table 5:** HTMT values

	CO	EE	MC	PO	RI	SC
CO						
EE	0.677					
MC	0.672	0.675				
PO	0.728	0.605	0.610			
RI	0.321	0.423	0.455	0.408		
SC	0.540	0.521	0.701	0.491	0.424	

MC=Main Contractor, SC=Subcontractor, PO=Project Owner, CO=Project Consultant, EE=External Environment, and RI=Partnering Relationship.

Table 6 presents the path coefficients of the model. The R-Square value of 0.246 shows that all the five independent variables, namely project consultant-related, main contractor-related, subcontractor-related, project owner-related, and environmental-related problem factors could explain about 25% of the variance in the partnering relationship. However, only the cluster of factors related to the subcontractor significantly affected the quality of the partnering relationship.

**Table 6:** Path coefficients

Hypothesis	Relationship	Std beta	Std Error	t-values	p-values	BCI LL	BCI UL	Decision
H1	MC -> RI	-0.157	0.091	1.715	0.087	-0.340	0.017	Not supported
H2	SC -> RI	-0.183	0.089	2.060	0.040	-0.363	-0.016	Supported
H3	PO -> RI	-0.173	0.113	1.523	0.128	-0.394	0.046	Not supported
H4	CO -> RI	0.063	0.106	0.598	0.550	-0.112	0.299	Not supported
H5	EE -> RI	-0.177	0.104	1.692	0.091	-0.396	0.012	Not supported

R^2^ = 0.246, Adjusted R^2^ = 0.214. MC=Main Contractor, SC=Subcontractor, PO=Project Owner, CO=Project Consultant, EE=External Environment, and RI=Partnering Relationship.

## 2 Experimental design, materials and methods

The research utilized a quantitative approach, in which a self-administered online questionnaire was developed to incorporate the problem factors affecting the quality of the partnering relationship between the main contractors and subcontractors. The problem factors were adopted from the framework of [6]. The measurement items used a five-point Likert scale with 1= “strongly disagree” and 5 = “strongly agree”. The questionnaire for this dataset is attached as a supplementary file. Questionnaire was distributed via Google Forms to the construction managers and engineers of project contractors and subcontractors in Kuala Lumpur, Shanghai and New Delhi. Before the online survey was conducted, the respondent was informed on the participation of the survey is based on voluntary basis. The Confidential Agreement was attached with the questionnaire when the questionnaire was distributed to the respondents. Purposive sampling was used as the questionnaire targeted project practitioners such as project managers, site managers, and engineers. A total of 144 completed questionnaires were received. However, 20 of these returned questionnaires were not usable as they were completed by non-target respondents, yielding the final sample size of 124. This sample size of 124 exceeded the minimum sample size of 92 generated from G*Power with the effect size of 0.15, significant level of 0.05, power of 0.80, and five predictors. Following the approach used by [6], the Relative Importance Index method was used to rank the problem factors as perceived by the main contractors and subcontractors. Partial Least Squares-Structural Equation Modelling (PLS-SEM) was used to check the convergent validity and discriminant validity of the data via measurement model assessment while structural model assessment was performed to verify the cluster of factors that plays a role in explaining the MC-SC partnering relationship.

## Ethics statements

Before the commencement of the project, the Institutional Ethics Committee (IEC) of UCSI University had granted the approvals for the data collection (IEC-2022-FBM-081). The self-administered survey was carried out in complete anonymity for the respondents. No sensitive or private data that could be used to identify the respondents was gathered. Before the online survey was conducted, the respondents' consent to participate in the survey was obtained.

## CRediT author statement

Eric Yao: Conceptualization, Methodology, Formal analysis, Writing – original draft, Writing –review & editing; Yet-Mee Lim: Conceptualization, Methodology, Formal analysis, Writing –original draft, Writing – review & editing; Choi-Meng Leong: Conceptualization, Methodology, Formal analysis, Writing – original draft, Writing – review & editing; Wai-Chow Lee: Conceptualization, Methodology, Formal analysis, Writing – original draft, Writing – review & editing; Chuen-Khee Pek: Conceptualization, Methodology, Formal analysis, Writing – original draft, Writing – review & editing.

## Declaration of Competing Interest

The authors declare that they have no known competing financial interests or personal relationships that could have appeared to influence the work reported in this paper.

## Data Availability

Dataset Describing Problem Factors of Partnering Relationships in the Malaysian ConstructionIndustry (Original data) (Mendeley Data)
